# The Role of Telemedicine in Follow-Up for Cardiovascular Hospitalizations∗

**DOI:** 10.1016/j.jacadv.2022.100154

**Published:** 2022-12-30

**Authors:** Srinath Adusumalli, Eric Bressman, Lauren Sinnenberg

**Affiliations:** a CVS Health, Woonsocket, Rhode Island, USA; b Leonard Davis Institute of Health Economics, University of Pennsylvania, Philadelphia, Pennsylvania, USA; c Corporal Michael J. Crescenz VA Medical Center, Philadelphia, Pennsylvania, USA; d Division of Cardiovascular Medicine, Perelman School of Medicine, University of Pennsylvania, Philadelphia, Pennsylvania, USA; e Department of Healthcare Management, The Wharton School, University of Pennsylvania, Philadelphia, Pennsylvania, USA; f Division of General Internal Medicine, Perelman School of Medicine, University of Pennsylvania, Philadelphia, Pennsylvania, USA

**Keywords:** telemedicine, transition of care

**Figure:**
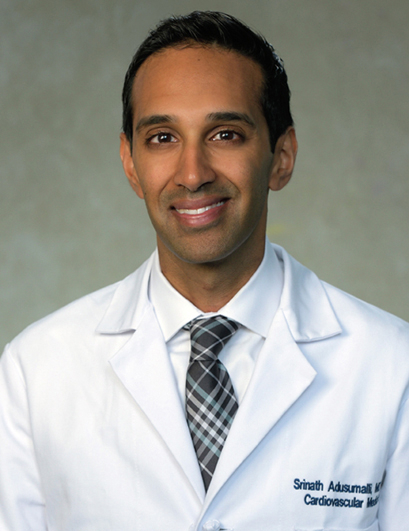

The field of telemedicine has experienced a dramatic shift, due in large part to the need for physical distancing and expanded payer reimbursement during the COVID-19 pandemic. The volume of telemedicine visits for outpatient care peaked during the early stages of the pandemic but remains at levels several orders of magnitude higher than that prior to the pandemic.^1^ Technology has evolved over the course of the pandemic as health systems and the broader health care delivery industry have worked to adapt to this new context of care. Telemedicine platforms and their user interfaces have improved significantly, and the adoption of remote examination devices as well as remote patient monitoring has grown. While telemedical care delivery has increasingly become a part of our health care ecosystem, there remain open questions about how telehealth has impacted care for patients with cardiovascular diseases.

In this issue of *JACC: Advances*, Tang et al^2^ make an important contribution to the evidence base for the merits of telemedicine in cardiovascular care through the lens of frequency and timeliness of posthospitalization outpatient follow-up appointments for acute cardiovascular hospital encounters. Postdischarge follow-up rates for all cardiovascular hospital encounters within a single, large, U.S., multihospital academic health system were examined before and immediately after the peak of the COVID-19 pandemic. The authors demonstrated a substantial increase in the use of telemedicine visits (both video and audio-only) for follow-up care after hospital admissions for an acute cardiovascular condition in the pandemic “steady state” compared to the seasonally matched time period prior to the pandemic. Nearly half of the follow-ups that occurred during this period were performed remotely. When adjusted for patient and encounter characteristics, they demonstrated that a greater proportion of patients had postdischarge follow-up within 14 days of a heart failure (HF) discharge in the “steady-state” pandemic period of 2020 compared to 2019. This appeared to be driven by more timely follow-up appointments for the HF patients than the prepandemic period. No differences were seen in patients following a discharge for other acute cardiovascular conditions including acute coronary syndrome, valvular disease, or arrhythmia. There were also no differences in 30-day unplanned readmissions between the time periods for any acute cardiovascular condition.

This study adds to our understanding of the role of telehealth in the transitional care of cardiovascular conditions, particularly as it pertains to access to care in the postdischarge period. This is part of a growing body of literature that expanded availability of telemedicine has promoted access to care during this critical recovery period.^3^^,^^4^ There are, however, limitations to this work.

Tang et al^2^ included patients within a single, large U.S.-based health system, and their population was further narrowed to only those who had established, recent outpatient care within that health system prior to their hospitalization. As noted by the authors, this health system cares for a lower proportion of non-White and higher proportion of patients from high-income zip codes as compared to the U.S. population, raising concerns about the generalizability of these findings, particularly through a health equity lens. While there is substantial emerging evidence that telemedicine programs may narrow existing inequities in care, contrary to concerns early in the pandemic, it remains critical that studies of telemedical care delivery include patients who are at risk of decreased access to care via digital channels (eg, non-English speakers, patients with lower income).^4^, ^5^, ^6^ These data are vital for health systems seeking to build telemedicine programs with equitable access as a guiding principle.^7^

While Tang et al^2^ demonstrate both that there was an increase in volume of telehealth encounters between the 2 time periods and that there was an increase in timely HF follow-up care, a causal relationship was not established through this work. It is possible that there were other drivers for the improvement of timeliness of HF follow-up care during this time. For example, perhaps there was a decrease in routine patient follow-ups during this period due to physical distancing, creating more postdischarge appointment availability. Future studies could work to clarify this link.

Interestingly, the increased follow-up rates demonstrated in this study were only seen among patients discharged after a HF hospitalization, not other acute cardiovascular conditions. Close follow-up after a HF hospitalization is essential, in part, due to a high 1-year mortality and a high 30-day readmission rate after HF hospitalization. However, out of those included, HF is also the condition that is most dependent on data that may require in-person interaction for acquisition, for example, the physical exam for volume status and laboratory tests for medication safety. While there has been increased interest in investigating the validity of remote examination for HF patients,^8^^,^^9^ the effectiveness of the remote HF follow-up visit compared to an in-person appointment has not yet been fully demonstrated. While the timeliness of the follow-up is important, the quality of the assessments performed during the encounter is similarly critical. The authors did not comment on whether the patients who had remote follow-up appointments had access to tools for remote examination and monitoring including blood pressure cuffs, scales, and pulse oximetry. Access to these data points, and understanding their impact on care delivery, is essential for clinicians seeking to assess patient status and make management decisions via telemedicine.

As a surrogate for quality, the authors assessed 30-day readmission rates between the 2 time periods. While the similar rates of readmission between the time periods suggest that there may be a similar quality of care delivered via telehealth and in-person visits and that the authors adjusted for patient and encounter characteristics in their comparison, it is of course possible there are remaining confounders present when comparing the study periods. Future studies can assess adverse outcomes in patients randomized to each follow-up context.

In addition to assessing disease-specific outcomes, future work must be sure to include patient-reported outcomes. Qualitative and mixed-methods studies are needed to characterize the patient experience in telehealth and to better understand how the shifting reliance on remote care serves the patient as well as the health system. This information, combined with disease-specific data, could be utilized to create a framework to understand which types of patients and diseases would be amenable to remote care and which are best served by traditional in-person assessments (or a combination of both). Models of care must be developed that can match the right patient with the right care context at the right time and be fluid as these parameters shift.

Finally, this study primarily investigated follow-up visits and outcomes for acute cardiovascular care during the COVID-19 pandemic. While there are still ongoing COVID-specific considerations in 2022, the landscape of pandemic-related restrictions, as well as the quality and functionality of telemedicine systems, has changed drastically since this time. Patients' interest in remote visits may change as physical distancing norms shift. A similar analysis performed in the current era may have significantly different results.

With this study, Tang et al^2^ advanced the knowledge in the field of cardiovascular telemedical care delivery by demonstrating that the increased use of remote follow-ups was associated with improved access to care after HF hospitalization. Given the rise in the use of telemedicine and adjacent care delivery modalities through the COVID-19 pandemic, and their ongoing promise for patient experience and clinician efficiency, health systems that utilize this channel of care should continue to pursue rigorous observational, quasi-experimental, and experimental evaluations of telemedical care to demonstrate its effectiveness, safety, equity, and patient-centeredness in the context of outpatient cardiovascular care.

## Funding support and author disclosures

Dr Adusumalli is employed by CVS Health. All other authors have reported that they have no relationships relevant to the contents of this paper to disclose.

## References

[1] Patel S.Y., Mehrotra A., Huskamp H.A., Uscher-Pines L., Ganguli I., Barnett M.L. (2021). Trends in outpatient care delivery and telemedicine during the COVID-19 pandemic in the US. JAMA Intern Med.

[2] Tang M., Holmgren A.J., McElrath E.E. (2022). Investigating the association between telemedicine use and timely follow-up care after acute cardiovascular hospital encounters. JACC Adv.

[3] Bressman E., Russo A., Werner R.M. (2021). Trends in outpatient care and use of telemedicine after hospital discharge in a large commercially insured population. JAMA Health Forum.

[4] Bressman E., Werner R.M., Childs C., Albrecht A., Myers J.S., Adusumalli S. (2022). Association of telemedicine with primary care appointment access after hospital discharge. J Gen Intern Med.

[5] Eberly L.A., Khatana S.A.M., Nathan A.S. (2020). Telemedicine outpatient cardiovascular care during the COVID-19 pandemic: bridging or opening the digital divide?. Circulation.

[6] Fear K., Hochreiter C., Hasselberg M.J. (2022). Busting three myths about the impact of telemedicine parity. NEJM Catal Innov Care Deliv.

[7] Samuels-Kalow M., Jaffe T., Zachrison K. (2021). Digital disparities: designing telemedicine systems with a health equity aim. Emerg Med J.

[8] Mohebali D., Kittleson M.M. (2021). Remote monitoring in heart failure: current and emerging technologies in the context of the pandemic. Heart.

[9] Drazner M.H., Kelly S.A., Schesing K.B., Thibodeau J.T., Ayers C.R., Drazner M.H. (2020). Feasibility of remote video assessment of jugular venous pressure and implications for telehealth. JAMA Cardiol.

